# Clinical Holistic Health: Advanced Tools for Holistic Medicine

**DOI:** 10.1100/tsw.2006.336

**Published:** 2006-02-24

**Authors:** Søren Ventegodt, Birgitte Clausen, May Lyck Nielsen, Joav Merrick

**Affiliations:** ^1^ Nordic School of Holistic Medicine, Teglgårdstræde 4-8, DK-1452 Copenhagen K, Denmark; ^2^ Quality of Life Research Clinic, Teglgårdstræde 4-8, DK-1452 Copenhagen K, Denmark; ^3^ Quality of Life Research Center, Teglgårdstræde 4-8, DK-1452 Copenhagen K, Denmark; ^4^ Scandinavian Foundation for Holistic Medicine, Sandvika, Norway; ^5^ Vejlby Lokalcenter, Vejlby, Denmark; ^6^ National Institute of Child Health and Human Development, Ben Gurion University of the Negev, Beer-Sheva, Israel; ^7^ Center for Multidisciplinary Research in Aging, Ben Gurion University of the Negev, Beer-Sheva, Israel; ^8^ Zusman Child Development Center, Ben Gurion University of the Negev, Beer-Sheva, Israel; ^9^ Faculty of Health Sciences, Ben Gurion University of the Negev, Beer-Sheva, Israel; ^10^ Office of the Medical Director, Division for Mental Retardation, Ministry of Social Affairs, Jerusalem, Israel

**Keywords:** quality of life, QOL, philosophy, human development, holistic medicine, public health, medical chart, holistic healing, holistic existential therapy, Denmark, Israel

## Abstract

According to holistic medical theory, the patient will heal when old painful moments, the traumatic events of life that are often called “gestalts”, are integrated in the present “now”. The advanced holistic physicians expanded toolbox has many different tools to induce this healing, some that are more dangerous and potentially traumatic than others. The more intense the therapeutic technique, the more emotional energy will be released and contained in the session, but the higher also is the risk for the therapist to lose control of the session and lose the patient to his or her own dark side. To avoid harming the patient must be the highest priority in holistic existential therapy, making sufficient education and training an issue of highest importance. The concept of “stepping up” the therapy by using more and more “dramatic” methods to get access to repressed emotions and events has led us to a “therapeutic staircase” with ten steps: (1) establishing the relationship; (2) establishing intimacy, trust, and confidentiality; (3) giving support and holding; (4) taking the patient into the process of physical, emotional, and mental healing; (5) social healing of being in the family; (6) spiritual healing — returning to the abstract wholeness of the soul; (7) healing the informational layer of the body; (8) healing the three fundamental dimensions of existence: love, power, and sexuality in a direct way using, among other techniques, “controlled violence” and “acupressure through the vagina”; (9) mind-expanding and consciousness-transformative techniques like psychotropic drugs; and (10) techniques transgressing the patient's borders and, therefore, often traumatizing (for instance, the use of force against the will of the patient).We believe that the systematic use of the staircase will greatly improve the power and efficiency of holistic medicine for the patient and we invite a broad cooperation in scientifically testing the efficiency of the advanced holistic medical toolbox on the many chronic patients in need of a cure. The level-8 tools can traumatize the patient if used incorrectly. Some of the level 9 tools and most of the level-10 tools can be severely traumatising for the patient, even when used correctly, so there must be compelling reasons for using them, and the patient must know, understand, and accept the risk before the onset of treatment.

## INTRODUCTION

In principle, the holistic healing of a person is a simple thing: an old and frozen "now" or "gestalt" that contains repressed and painful emotions from past life events needs to merge with the present, and when this process of integration of denied parts of existence is successfully done, the healing is completed[1,2,3,4]. In practice, the merging is often everything but simple. One of the strongest reasons is that the patient really does not want to suffer again (because he has to deal with old "hidden" pain), so the most fundamental principle in clinical holistic medicine is to work with the patient's resistance towards feeling, remembering, and confronting the content of the subconscious[5]. In principle, the holistic physician or therapist can do this work in two opposite directions and either go with the resistance or go against it. In therapeutic practice, this is always "a dance", one step in the one direction and one step in the other. When you go with the resistance, you comfort your patient and win sympathy; when you go against it, you raise the patient's consciousness, awareness, and presence. When you go with your patient's true self, you go against the resistance and when you go with the resistance, you go against the patient's true self. Unfortunately, because of the patient's repression of the true self, going with the deepest emotional layer of the patient is often going against the more superficial layer of the patient's existence and, paradoxically, this is often experienced by the patient as going against him or her — hence the dance.

As the therapy progresses successfully, earlier experiences of the patient's personal history (the still old repressed painful gestalt) appear in the therapeutic sessions. As therapy regresses the patient to earlier in life, more and more abstract existential problems are confronted and the philosophy of life of the patient are gradually turning more positive and responsible[4], while the negative attitudes serve the purpose of justification of displacement of responsibility from self to the outer world. As the patient often spontaneously moves back in time during the therapy, integrating more and more of the repressed material, the "energy" of the gestalts is normally raising. The reason for this paradoxical situation is that the patient will confront still stronger emotional pains as the therapy goes deeper due to the level of arousal and the intensity of emotions, which are generally higher if the traumas were from childhood. When the patient is back in early childhood, the emotional intensity is normally quite extreme (compare to Janov's "Primal Scream") compared to the emotional intensity of adults, and as the regression progresses further into the re-experience of the life in the womb[6]. These well-repressed traumas are often so intense that it takes several persons to support the patient to give enough holding for him or her to confront fully the extremely intense subconscious material, both pleasant and unpleasant[7].

## THE DARK SIDE OF THERAPY

To get a patient who is in need of care and attention to work in therapy is often quite easy. Physicians often work as therapists after only a few weeks of training, as we know it from young physicians entering psychiatry. Working with biographical and personal history, perception of self and reality, and similar issues is also often quite easy with a motivated patient. As the therapy goes deeper, the patient will reveal a higher and higher degree of resistance and the competence of the physician or therapist must rise accordingly to match the needs. When the therapy takes the patient into the deepest layers of the consciousness, the experiences often get quite disturbing for the patient. The emotional pain will often be overwhelming and the therapist will then meet the dark side, the shadow, of the patient and in this meeting, the therapist will often also meet his own shadow. In some cases, this shadow can materialize as directly evil towards the physician and others[8]. It can be shocking for the therapist who is only trying to help the patient from the best of intentions when the patient suddenly turns on the therapist with evil intentions. When the therapist uses strong therapeutic techniques to confront the shadow side of the patient, the patient cannot escape and all the negative aspects can then arrange themselves around an abstract center of evil, which in many ways is similar to the good essence of the person, which is his purpose of life, or life mission[9]. Confronting the patient in his negative, evil intended side can take form as the classical ritual of “exorcism”[8], where the patient is completely obsessed with “the devil” or the patient can enter into a psychotic state of mind lasting for minutes, hours, or days[10,11]. If the therapist is not experienced or confident with the holistic treatment of insanity, the therapist can be overwhelmed as the resistance of the patient “wins the game” and then the holistic therapy can be turned into traditional psychiatry with the danger of creating further trauma and without healing of the patient’s existence, which was the purpose of the therapy.

If the therapist is caught unprepared in the process of meeting the shadow side, which we call negative transference (often happening after a period of positive transference, where the patient has been into strong admiration or even secretly in love with the therapist), and working into the dangerous trap of counter-transference and suddenly being the weaker part, instead of staying strong, balanced, and in control of the session, the therapist can also be deeply hurt emotionally. If the therapist goes completely out of control and into emotionally driven, highly irrational behavior (it can happen when the therapist is strongly hit by what is happening), very unfortunate things can happen. Sometimes the patient will fight to leave the room, while the physician will physically hold him or her back. Afterwards the patient might complain that the therapeutic contract was violated, or even accuse the physician of violent or sexual abuse. Such an experience can be so embarrassing that it can tempt even a trained therapist to drop his whole carrier as a therapist.

So “the dance” of therapy, as it grows in intensity, can turn into a fight and a true nightmare, where the therapist loses all control and the patient’s dark side takes over the session. What normally happens in this situation is from a depth-psychological perspective that powerful gestalts of the therapist himself (his own inner conflicts) materialize during the therapy. The better the therapist knows himself, the farther the therapist has come in his own therapy, the farther into the depth of the ocean of consciousness the therapist himself has penetrated, the farther he/she can also take the patient. But everybody has their repressed emotions and every therapist must learn in order for severe errors, mistakes, and failures not to happen. Constant supervision and personal therapy is a must and lifelong supervision is strongly recommended. Over time, the therapist will normally, step by step, be more confidant and competent and able to use still stronger tools from the advanced holistic medical toolbox. Therefore, the holistic therapist should not expect to be able to use the most difficult tools for the first several years, since it takes a lot of time and experience to learn to lead the session at that speed and intensity. The maturity of the therapist must also be taken into consideration here.

## WHEN REGULAR THERAPY IS NOT ENOUGH

The holistic physician normally works with love, trust, holding (awareness, respect, care, acceptance, and acknowledgment), therapeutic touch, conversational therapy, and exercises intended to upgrade the philosophy of life of the patient, combined with the standard medical assessment and examination[12,13]. Except for a modest risk of verbal abuse and physical intimidation, these techniques must be considered safe for the patient if they are done correctly and according to a previous therapeutic contract. But these rather risk-free techniques are not always enough to make the patient heal. And failure is not really an option, as failure normally means the patient’s gradual or sudden loss of health, ability, and quality of life.

In the many cases where a mental or physical disease is not disappearing in spite of more superficial therapy, it is sometimes necessary to use techniques that help the patient to match the high levels of neural arousal and emotional intensity of the early traumas. Some patients with a more reflective nature will need a deeper process and some diseases like cancer often need a deeper process than a less-severe disease like arthritis. To fully rehabilitate the three most fundamental dimensions of existence (which according to our thinking are love, power, and sexuality[14,15,16]), the therapist will need to guide the patient into the deepest corners of the soul, mind, and spirit or life itself. This journey goes into the famous underworld and inferno of Dante (Dante Alighieri, 1265–1321)[17], which will take the patient through the most intense emotional and spiritual pains. Life is suffering, as Gautama Buddha (563–483 BCE) taught, and deep existential therapy often reveals this fundamental truth. Only when we let go of what we cling to, Buddha also said, the suffering will disappear. Letting go of what we cling to in our mind and life is essentially what existential holistic therapy is about.

In the situation where the patient is not healing because deeper existential layers need to be integrated, the physician is obliged to take the art of healing a step further. A "radical new cure" must now be invented for the patient and the means must be judged against the risks. The physician must deeply consider the old Hippocratic saying: "First do no harm". It is true that no physician can be expected to cure all patients, but still it is the duty of the physician — as long as the patient himself insists on fighting for his life — to do his best and continue to do so, until the day the battle is either definitely won or definitely lost. The physician must judge, in every case, if it is possible at all to cure the patient and if this is really within his reach as physician. As the outcome of any treatment is really unknown beforehand, because it is strongly dependent on the patient himself, the physician must also estimate a likelihood that the intended cure will help in order not to waste time and resources on a hopeless case.

**10 Border transgressing techniques**
**9 Mind expansion**
**8 Existential work**
**7 Energy work**
**6 Spiritual healing**
**5 Social healing**
**4 Emotional healing**
**3 Support**
**2 Intimacy**
**1 Relation**

**FIGURE 1.** The staircase of advanced tools for holistic medicine.

**TABLE 1**
**The Staircase of Increasingly Intense and Potentially Traumatic and Dangerous Holistic Medical Therapeutic Tools***

| **1 Relation** | **2 Intimacy** | **3 Support** | **4 Emotional healing** | **5 Social healing** |
|---|---|---|---|---|
| Love and acknowledgment; Talking about patient's biography | Winning the patient's trust; Music and art therapy; Dance and movements; Massage | Giving holding: awareness, respect, care, acceptance, and acknowledgemnt; Coaching; Time-line therapy | Taking the patient into the process of healing by touching, talking, and setting perspective; Intentional work (sweat lodge ritual) | Active work with projections; Mirroring good and evil in the patient; Working visibly with and against the patient's resistance; Group therapy, psychodrama, body dynamics; Sharing circle (native social rituals) |
| **6 Spritual healing** | **7 Energy work** | **8 Existential work** | **9 Mind expansion** | **10 Border transgressing** |
| Rehabilitation of life purpose, character, and coherence; Acceptance through touch; Soul-body-body-soul; Deep coherence between patient and therapist | Rising energy circles; Holistic breath work; Painful provocative body work; Sexual polarity work | "Controlled violence"; Acupressure through the vagina and anus; Direct sexual stimulation; "Controlled sexual abuse"; "Exorcism"; "Controlled fail" of the patient" | Psychotropic drugs; Substitute partners; Death-rebirth rituals; Mitote ritual; Ritual life burial; Killing and revival; Sundance ritual; Shamanism (i.e., materialization) | Sedating drugs, antipsychotic drugs; NCE; Use of force against patient's will; Institutionalization; Surgery in general; Mutilating rituals like hanging in the chest muscles (Native American procedures); [Direct sexual involvement] |

*(1) Love, (2) trust, (3) holding, (4) healing, (5) group therapy, (6) life purpose-character-coherence, (7) "energy" work, (8) cathartic work, (9) mind-expanding/ego-transformative techniques, (10) extreme (often traumatizing) techniques (see text).

When everything else has been tried, but the healing has not occurred and the physician still senses that there is more to be done, the holistic physician can — if he has the necessary qualifications such as training in medical ethics and in the different treatment techniques, combined with a sufficient level of personal development and sufficient courage — use the advanced tools of holistic medicine.

The advanced holistic physician's expanded toolbox contains powerful tools that can be organized into a staircase of the intensity of the therapeutic experience that they provoke and the level of expertise they take to master (see Fig. 1 and Table 1). The more intense a therapeutic technique, the more emotional energy will normally be contained in the session and the higher the risk for the therapist to lose control or lose the patient to the dark side, which can make the therapeutic session very traumatic and damaging. These induced problems can almost always be healed if the patient stays in the therapy, so the real risk is losing the patient because he or she completely drops out of the therapy.

As demonstrated throughout our many papers on clinical holistic medicine[10,12,13,18,19,20,21,22, 23,24,25,26,27,28,29,30,31,32,33,34,35,36,37,38,39,40,41,42,43,44,45,46,47,48,49], almost everything can be used as a tool, since only the imagination sets the limit. To induce the state of consciousness we call "being in the process of healing"[4], the physician (according to Yalom[50,51]) needs to invent a new cure for every patient. This ability to be imaginative, creative, and use whatever is necessary to induce the healing is the hallmark of the excellent therapist. Good intent, balanced action, and good results are definitely needed in holistic medicine. Giving up on your patient and not doing anything at all might be a bigger sin, in many cases, than doing your best as a holistic physician and still losing your patient. Still you need to use any tool only after careful consideration, respecting the golden rule never to use a tool more powerful and dangerous than necessary (compare that both in surgery and with chemotherapy the patient is risking death as a result of the treatment).

Almost everything in the world can be used as a tool, but as the physician lines up his tools, some tools are used naturally before others and some might be painfully out of reach because of lack of expertise or due to the laws of your country. The ranking of tools after intensity, danger, and needed expertise of the physician gives a “staircase” of advanced tools of holistic medicine; its function is to help the holistic physician to “step up” in the use of the techniques one level at a time.

Let us admit that therapy often is a little “messy” with the combination of a number of tools and techniques. To think of therapy as the clear-cut process of “walking the staircase” is too simple. Often, many of the steps are used in subtle and symbolic ways by the skilled therapist, i.e., hidden in jokes and ironic remarks. So this staircase is meant for education, training, and treatment strategy, and not to limit the flexibility and spontaneity of the therapy.

The concept of “stepping up” in the therapy by using more and more “dramatic” methods to get access to repressed emotions and events has led to the common notion of a “therapeutic staircase” with still stronger, more efficient, and more potentially dangerous traumatic methods of therapy (Fig. 1).

We have identified 10 steps of this staircase:

1. Is about establishing the relationship
2. Is about establishing intimacy, trust, and confidentiality
3. Is about giving support
4. Is about taking the patient into the process of physical, emotional, and mental healing
5. Is about social healing of being in the family
6. Is about spiritual healing – returning to the abstract wholeness of the soul
7. Is about healing the informational layer of the body (from old times called the ethereal layer)
8. Is about healing the three fundamental dimensions of existence: love, power, and sexuality in a direct way
9. Is mind-expanding and consciousness-transformative techniques, and
10. Techniques transgressing the borders of the patient and therefore often traumatizing, like using force and going against the will of the patient.

When the holistic physician or therapist masters one step, he can go on to training and using the techniques of the next step of the staircase. As step 10 is often traumatizing for the patient even with the best of physicians, it is generally advised that the holistic physician or therapist do not go there. When well mastered by the physician, steps 5–8 (9) can be used, when steps 1–4 do not help the patient sufficiently. The tools must be used one level at a time and each step implies an increasing risk for traumatizing the patient. Levels 8 and 9 often take many years of practice to master.

## Level 1: Relationship/Love

Loving your patient is the first step of helping, since only with love can you be at service in an unselfish, egoless way and love is the strongest resource in the art of helping another fellow human being. If love is not there, it cannot be forced or willed; maybe there is kindness and care, maybe an interest in the other person, which can be turned into a relationship. Just establishing a relationship is a powerful thing to do and in the acknowledgment of the other person’s personal history will you be able to help many wounds to be healed. It is important to say that love, in our understanding, originates from the urge to use your personal talents and give what you need to give to the world. Love is about living your personal mission.

### Level 2: Intimacy/Trust

When there is love, the patient's trust can be won, often little by little during time. With trust comes intimacy — physical, emotional, and mental closeness; then many things are possible, like massage, dance, art therapy, etc. Just learning how to trust and be intimate is a giant step forward for most patients, and their quality of life and self-esteem can be radically improved by the techniques of this level.

### Level 3: Support and Holding

When the patient trusts you, you can get permission to give holding; the five dimensions of this crucial existential support are (1) awareness to the mind, (2) respect for the patient's emotional space, (3) care for the body, (4) acceptance of gender and sexuality, and (5) acknowledgment of the soul and personal character. In giving these five qualities in a rich blend, you can help almost everybody to feel good and right.

### Level 4: Physical, Emotional, and Mental Healing

When the holding is there, the patient can get the support in the actual moment, which empowers him or her to go back to the old, emotionally painful and confront the repressed content of the traumas. Getting help now to process the old trauma is the secret of healing. To take the patient into the state of mind that we call "being in the process of healing" is what holistic medicine basically is about[4]. To get the patient into this state is really a question of intention; both the physician and the patient must intend that the healing will take place and "the bubble" the patient is isolated in must be open from the inside and the outside at the same time, as the shamanistic tradition claims[52].

### Level 5: Social Healing — Healing the Being in the Family

This level is about healing the relationship with the group and the family, where using a group for this kind of healing is a must. The Native Americans had their sharing circle and the talking stick; today we have the holistic existential group therapeutic process[7]. In the group, everybody can watch everybody, and one great advantage of this kind of work compared to individual therapy is that the process of working with or against the resistance becomes obvious to every member of the group. This makes it possible to help the member to watch his own projective mechanism of the consciousness, helping him or her to assume responsibility for the unconscious attitudes, the "color of the glasses of the spectacles" so to speak. This makes it possible for the therapist to mirror the patients in the group effectively, effectively helping the patients to realize their own idiosyncrasies, blind spots, and neurotic survival patterns.

### Level 6: Spiritual Healing — Healing the Abstract Wholeness of the Patient

On this level, the therapist must use his ability to sense the purpose of life[6,9] and the physical, mental, and spiritual character of the patient[7]. The purpose of life, or the life mission, is the core talent of this person, and happiness is about using this talent to be of value to the world. Other supporting talents surround the core talent and when claiming them, yet another series of tertiary talents comes into use. Being gifted and contributing to the persons dear to him or her and to society at large rehabilitates the existential coherence, the deep feeling of connectedness and belonging, which we long for deepest in our hearts. Rehabilitating the spiritual side of the patient is really allowing the patient to dig deeply into the hidden resources for healing him or her. Unfortunately, the ability to use the abstract sense necessary to master this level takes a lot of practice and time, often years. It develops as you obtain coherence with the outer world yourself, as a product of your own successful personal development. As you find this coherence, you will notice that you can connect soul to soul with your patient through your body and the body of the patient. When you take this skill into the sexual area and give acceptance to the body, the organs, the gender, and the sexuality of the patient, you master the technique called “acceptance through touch”[13]. Touching may simply be just placing your hand on the patient’s body.

## Level 7: Healing the Informational System of the Body (Ethereal Healing)

Consciousness meets the body in a peculiar way, creating what is often experienced as “circles of energy”, the different qualities of the body and mind being sensed as circulating sexual, emotional, mental, and spiritual energy (see [16] for an overview of the qualities). Raising these subjective circles of “energy” is called “working with the energy” and it really is difficult to describe what is going on in this work, as it is about supporting the patient in exploring all the hidden qualities of body, mind, and spirit. Often breathing is involved in this work, with holotropic breath work[53] as a fine example of this kind of energy work. It often helps the patient to integrate very early gestalts and spontaneous regression into the womb is normal in this kind of work. Some patients recall earlier incarnations, especially if the physician is open for this. Working with the energies of the body often leads to recollection of extremely painful memories from early life. Also, intense sexual energies are often awakened and training the patient to be a male/female pole in the universe is a part of the successful balancing of the patient’s energy. This level of biological information is poorly understood by contemporary science and for the last 2 centuries, occult research has been carried out referring to this layer of the human being as the “ethereal body”[54].

## Level 8: Direct Existential Healing of Love, Power, and Sexuality

According to the theory of talent, there are three fundamental dimensions of existence: love, power, and sexuality. These dimensions can be confronted en bloc, which gives overwhelming and extremely intense experiences in the therapy. When all the evil sums up to the essence of the shadow, the person manifests his evil alter-ego and it really looks like he is obsessed by Satan; hence the name “exorcism” for this tool. When a person has been violently abused throughout his childhood, anger can be so repressed that only hitting him again can release it. This can be used as the therapeutic technique originally developed by the famous founder of gestalt therapy, Perls[3], and we call this method “controlled violence”. Actually, every time the therapist goes against the resistance, there is an element of controlled violence toward the patient’s emotions, who often reacts as hurt and offended. Every time the therapist goes against the patient’s true self, there is an element of controlled violence against the patient’s soul. But violation of emotions and the soul is often not seen and is widely accepted. Violating the body physically by beating it (with open hand, not to cause any harm though) is seen by many people as unacceptably violent. From a theoretical analysis, there is really no difference; it is all controlled violence.

Another set of very strong and efficient techniques at this level is the technique’s relation to sexuality. The therapist can work against the resistance and with the patient by directly stimulating the patient sexually, which is a seldom-used technique. More often, the therapist will use a formalized technique like acupressure through the vagina to raise the energies in the pelvic area[55] or to confront repressed material connected to sexual abuse or neglect by stimulating the relevant tender points in the genitals and deep pelvic area reached through the anus and the vagina. The last technique has an aspect related to controlled violence and this kind of work is called “controlled sexual abuse”, as the patient in this kind of healing often will find the old painful emotions from the trauma in the present moment, not in the past. This seems to be a general rule of all high-energy traumas: the higher the energy and the more intense the emotional pain, the higher the likelihood for the trauma to manifest itself in present time, during life or in therapy. This means that you, as a therapist, should not always expect a child rape trauma to be manifested by the patient as their child rape trauma, but sometimes as a brand new experience of being abused in present time by the physician in the clinic, because of the transferences. The only way that the therapist can survive this legally is to address the problem directly and to make a therapeutic contract of "controlled sexual abuse", for the trauma to reappear in the session under controlled conditions and not allowing the patient to get away with the transference of the old, extremely painful material[47,48,49]. This kind of work takes a high level of expertise and years of practice to master. It must always be done with supervision, to be completely sure that the physician is not involving his own shadow in this kind of work. If the physician subconsciously is engaging in countertransference here, it can be very traumatic for the patient.

The most painful and difficult of the tools on this level is the "controlled fail" of the patient. There is hardly a patient who has no extremely severe wounds on their soul from early childhood, as it really is impossible to be a perfect parent. In some cases, just 30 minutes of mental distraction or physical absence can be experienced as a complete loss of both parents by a sensitive and vulnerable child. Some patients are worse off, because as children they experienced systematic fail from their parents. Often, they had the role as parents for their own parents from early childhood and to compensate for this, they developed a tendency to cling and adhere to finally obtain the love and contact they needed. In theory, this failure should be easy to alleviate, but as love is what is most important for us as human beings, systematically not getting the love we need and fight for throughout our childhood is giving such a traumatic series of emotionally painful wounds, that the moment the therapist intends to give his love, the resistance of the patient will be so intense that no love can be received. So going with the resistance is the only way to proceed and this means ignoring and abandoning the patient, while he or she is in the therapeutic session. This really is a paradox: the patient is paying for therapy and nothing is happening, no, less than nothing! Only the philosophically highly developed patient will understand what is going on and even this mental understanding will not help. This is as terrible as it gets, since this is sheer hell raised once again. But it is not really happening in the present time in the therapy, and a normal sound person will not enter into deep process of holistic healing feeling emotionally completely destroyed just from being ignored by another person. But these patients will. As the therapist can easily feel the transference, he must now avoid getting into countertransference and starting to feel evil himself.

The technologies on this level are highly efficient, yet they are just drills derived from the inner logic of the therapeutic process of holistic healing. In the hand of an untrained and poorly developed therapist, these are the cruel tools of torture and abuse that finally gives him the dark power over another person that his own evil shadow side has longed for his whole life. It is really easy to be tricked into the dark side when using the tools of level 8, so never start using level-8 tools without intense supervision and coaching by an experienced holistic physician who has mastered this level himself.

## Level 9: Mind-Expanding and Consciousness-Transformative Techniques

If level 8 was difficult, then level 9 is an art that really cannot be mastered without perfect mastery of the tools of level 8. One of the techniques of this level is being a substitute partner. To give yourself to this process of pairing up with a sexually dysfunctional person with the only purpose of healing their sexuality takes a rare kind of devotion. When it is done professionally and according to a contract (which normally implies only seeing each other for 14 days or so), it really works wonders for the patients[56,57].

Some of the techniques in this group are so difficult that in the premodern societies they were performed by a shaman or high priest, one of the highest developed persons in the tribe, who had devoted their whole life to this kind of practice and service. This is very much still the case. You can only develop the mastery of the skills of level 9 by being completely devoted to this kind of work for decades, unless you have a very special gift for it. Few students have. Gifted or not, you need to be a trained by a master in these techniques for years before you can do them on your own.

Some of the tools that demand this kind of mastery are the healing rituals, mostly carried out by Native Americans and other premodern cultures as extremely intense rituals, taking you all the way down to the core of your existence. One ritual takes you through a subjective experience of death and rebirth, while others makes your worst nightmare come through in the form of a life burial, to (almost) die from suffocation alone in darkness (used for integrating some of the most terrible fetal experiences, compare Stanislav Grof's BPM2[6]), and thereafter miraculously come back to life as your true self that you felt you had lost forever. To master such rituals takes the most loving and empathic kind of therapist, who minutely can read the state of mind and observe all changes in the patient's consciousness accurately enough throughout every moment of the whole ritual to meet the needs for the healing the patient. It takes a therapist who is completely familiar with the whole range of experiences of ego-death and personal transformation. This competence is only slowly developed through supervised training and personal experience throughout years.

Other tools of the ninth level that take similar mastery are the use of psychotropic drugs in therapy. In many ways, this is a lost art, but it has been extremely widely used, as most premodern cultures used them for millenniums. The word for medicine is the same as the word for the peyote cactus in many native North American tribes[58,59,60]. Other tribes have used the fungi of the species *Psilopsybe* (containing psilosybine) and the cactus called San Pedro (*Trichocereus pachanoi*) that, like peyote, contains mescaline as its major active substance. The liana called ayahuasca (*Banisteriopsis caapi*) has been used in South America, while other cultures like the ancient Egyptians used an LSD-like alkaloid derived from the Ergot of Rye, a plant disease caused by the fungus *Claviceps purpurea*[61]. All these substances contain psychotropic (mind-expanding) drugs or hallucinogens, but with a very different profile from the recreational drugs in popular use among young people all over the world today[62] like ecstasy, cocaine, and amphetamines, which have a strong CNS-stimulating effects in addition to more modest, mind-expanding qualities.

The purpose of the use of the mescaline-cacti among Native Americans is to bring the patient to a state of consciousness where he can realize how he makes himself ill by not living in accordance with the deep self[58] or, in our interpretation, with the true human character and the purpose of life[63]. This makes the fairly mysterious native medicine often completely incomprehensible due to the use of massive symbolism, which is very difficult to understand. We are proud to say that the consciousness-based medicine we have developed normally does not use any kinds of drugs, as this has not been necessary because of highly efficient therapeutic techniques and strategies. Unfortunately, very sick cancer patients and other terminal or hopelessly ill patients with very limited resources that we are trying to help seem to need stronger tools, which makes the use of hallucinogenic drugs relevant. The use of hallucinogenic drugs might be relevant in holistic medicine when patients only have very limited time and resources or are close to dying, i.e., from cancer with metastasis to lung, liver, or other vital organs, but the use is controversial.

Sometimes, the patients ask if such drugs would be useful for them and the answer is that without a proper therapeutic setting to support the process of healing, it will probably be a waste of time and not beneficial, or could even be harmful. Sometimes, the patients have been offered the drugs before. It often happens that travelers to the Amazon jungle are offered Ayahuasca by the local medicine man (some talk about "Ayahuasca tourism"). In such a case, it can be very difficult to help a patient further integrate the experience without using the drug again and only one such unintegrated experience of maximal intensity can be quite invalidating. If a hallucinogenic drug is to be used in the advanced or experimental holistic clinic, the most tested and traditionally most successful are without doubt mescaline[58,59,60,61], psilosybine[61,63,64,65], LSD-25[61,64,65], and Ayahuasca[66]. The effect of mescaline is the mildest, the easiest to control, and, thus, the less dangerous to use in a clinical setting, while psilosybine is stronger and LDS-25 and Ayahuasca are traditionally regarded as the strongest hallucinogens.

It is very important to understand why previous attempts to use hallucinogenic drugs as medicine by western physicians have mostly failed and why the drug experience can be damaging[67]. It takes a high level of expertise to lead the patient into the holistic state of healing in general and presumably an even higher expertise to do this when the patient is on a psychotropic drug or any other drug that affects brain activity. The steps of "loving" the patient, winning trust, getting permission to give holding, and finally letting the patient surrender into the process of holistic healing is mandatory. A psychotropic drug can never do more than facilitate a process.

We have visited the seven tribes of the Round Wally of Northern California recently and could not find any medicine man that was able to bring his patients into the state of holistic healing using peyote or a similar substance. We met one medicine man who told us that the tradition of holistic healing using the peyote was sadly lost in his tribe after the movement of the tribe from its original habitat by the U.S. government almost 100 years ago. With the decay of the Native American culture, the tribes are not able to keep their old medical tradition alive and the wisdom of holistic medicine accumulated through the centuries is sadly lost.

While both the European witches and the Native American medicine men have used hallucinogenic drugs for centuries (like the cactus peyote, together with psilosybine-containing mushrooms, scopolamine-containing plants of the family Datura, and many other potent hallucinogens), the modern physician has refused to use such drugs in the service of healing his patient. From the inner logic of the therapeutic staircase, the reasons for using these drugs are obvious: they intensify the therapy and help the patient go back to more intense and emotionally painful material. In a way, these drugs are easy to use, you just give them to the patient; the patient can even self-administer them without a competent therapist present. In most cases, this will work out fine as our mind always makes a calculation of the resources available and never allows us to go deeper than we have resources to. This is very much also the case with most of the drugs (mescaline, psilosybine), except for the strongest of the hallucinogens (the LSD-like). Even with LSD-25, your mind will give you a lot of protection, making unbearably bad trips very rare. Without mastering the previous levels of the ladder, a therapist cannot make use of the full power of the psychotropic drugs, as you must be able to read intent and be able to follow your patients into their altered states of consciousness, to understand what is going on with them and what they need for their healing along the way. When on the drugs, the patient's shadow side is most likely to take over if not well guided and the therapy will run off the track, and this is why the use of the hallucinogenic drugs can really harm the patient. The intensity of the drug experience means that drug-induced traumas often can only be processed on the same drug; if a therapist cannot control the session with a patient on such a drug, his chances to repeat it and succeed are small. Therefore do not do this kind of therapy without the guidance, training, and supervision of a master of the level-9 techniques. Unfortunately, as there are not so many shamans left in the world, it seems that we have some experimental research to do to obtain the mastery of level 9.

The use of psilosybine-containing mushrooms, which we used for our own research purpose, was made illegal around 2000 in Denmark. Since then, we have been without a legal potent hallucinogen; for legal reasons we have not used such drugs in the clinic and are therefore unable to help our cancer patients and others who need it. As we are convinced that this tool is highly efficient in holistic healing in some special cases where the lower-level tools have failed, we believe that they should be taken into use by the holistic physicians again. Most importantly, we believe the level-9 tools to be far less traumatizing than the often-used level-10 tools, the use of which might be reduced if the lower-level tools were used more. This could be of major benefit for the patients.

## Level 10: Techniques that Transgress the Patient's Personal Borders (Often Traumatizing)

It is obvious from Fig. 1 that many of the level-10 tools are in frequent use in modern-day medicine. When the use of moderate power does not work, more powerful tools are frequently used; these are techniques like brute force against the patients will, sedating drugs, institutionalization, and even imprisonment and severe invalidations of basic human rights in some countries, even though most researchers agree that they are often severely traumatizing the patient. The reasons why they are in use are, of course, the failure to help the patients with less-radical means, or the failure of confidence in the lower steps, making the physician skip directly to level 10. Most of the steps of the staircase are not taken into use by many modern physicians in the western world; sadly, often only level-1 techniques are tried before going to level 10 in many highly developed countries.

It seems that the art of holistic healing using the first 9 steps are sadly lost in many countries and instead of practicing love and healing the patient, brute force is used. We hope that reintroducing the therapeutic staircase will inspire many physicians and therapists to use the less-powerful and less-traumatizing means of the lower steps to heal their patients in the future.

One other potent tool, which is often used by modern-day therapists, sometimes motivated by love, sometimes motivated by abusive intentions, is direct sexual involvement with the patient. While such an endeavor has been argued strongly against ever since Hippocrates, it seems that there has been a constant decay of some therapist’s ethics throughout the last century. One female patient around 25 years old, with a personality disturbance, told us about at least four different therapists who had abused her sexually.

The reason for not having sex with a patient is that this behavior completely disturbs the relationship, turning it upside down, giving the power to the patient and making therapy impossible. As the patient often loves and admires the therapist, this can also be seen as abuse of the power of the therapist and, all too often, the girl is left behind as the therapist moves on to abusing yet another patient and thereby failing the patient and their profession.

Direct sexual involvement with the patient is not the right thing to do from a therapeutic perspective, except in one special occasion: when the patient and the therapist have fallen mutually in love, when the therapy has gone definitely stock and every session from therapeutic perspective has become a sheer waste of time, and when neither of them has obligations towards others, which forces them to fail each other at a later occasion. As the laws of almost all countries forbid the physician to have sex with a patient, the therapy must then be formally ended, there must be an appropriate gap in time from ending it to engaging sexually, and the relationship must also be successfully redefined before direct sexual involvement is possible. Because of the negative view of such a relationship from society, it is still recommended to keep such an engagement within the framework of the tool of a substitute partner. Direct sexual engagement with a patient is a good example of a level-10 tool, often having a traumatizing effect. The level-10 tools are in general so traumatizing, in spite of all good intentions, that they cannot be recommended in the holistic medical clinic; although the use of them cannot always be completely avoided.

## USING THE STAIRCASE FOR TRAINING THE HOLISTIC PHYSICIAN

The training of the holistic physician is difficult, since the only way to learn is to practice and do it. Learning by doing means that in the beginning, the student will make every possible mistake and error, and the coach must be very involved and close to correct the errors and mistakes before they lead to any serious consequence. In practice, it is often very easy for a skilled therapist to correct the errors if the student loses control of the session; the senior therapist will take over and then reinsert the student very much the way a new driver learns to drive a car. Interestingly in therapy, the situation with the patient is, as a rule, better after a failure and a recovery than before the failure. This happy situation is a result of the mutual learning of the patient and the physician or student. Not being willing to learn from mistakes, and therefore hiding them from oneself or others, is the most dangerous behavior a trainee or physician can have. Unfortunately, many university hospitals have little mercy with physicians and students who make mistakes, which creates an environment of fear and of hiding. The most important thing in good training is the rule that all mistakes are allowed, but only once. In biomedicine, when a mistake with drugs and surgery is often fatal, this kind of freedom is more difficult to give students; in the holistic clinic, the most difficult of tools are hardly ever fatal.

Complete familiarity and mastery of one level of techniques leads naturally to the next and after many years of training and practice, all the levels can be used. Using the level-10 tools is something even the most skilled holistic therapist only will do hesitatingly. It is of utmost importance to know how to use these tools to use them wisely and avoid traumatization. If force is necessary, or if the use of strong sedatives and antipsychotic drugs are necessary (i.e., because the patient is trying to kill somebody or trying to commit suicide), the physician must know exactly how to react concerning force or drugs used. If the physician has fallen in love with a patient bringing therapy to an end, he or she must know how to deal with this extremely difficult situation by finding a supervisor for support, avoiding sexual contact before the roles are sufficiently redefined, and balancing the relationship, so that this can be considered safe for the patient. Not knowing how to use these tools can be very dangerous for both the patient and the physician. Let us underline that we believe that a sexual relationship between a physician and his patient is to be avoided at all times, also after the treatment is formally terminated.

## DISCUSSION

Since Hippocrates, one of the most important principles in medicine has been “first do no harm”. Medical ethics is, therefore, the primary concern of every holistic physician when using advanced and emotionally intense tools of holistic medicine with the potential to afflict further traumas instead of helping. Often in the clinical practice, even a severe mistake can fortunately be corrected, as traumas induced by therapy can be healed in the same way as every other trauma. On the other hand, it will take a therapeutic session of similar intensity as the damaging session to heal the wound and sometimes this is not possible as the patient is not willing to give it another try if the first session was very painful and scary.

In daily practice, this means that *every procedure must be justified* in two ways: (1) no procedure should be carried out if one with less risk and less intensity of the impact —emotionally, physically, and otherwise — can do the job and (2) what is likely to be won for the patient by using this procedure should be much more than will likely be lost. The patient must always be informed of the risk involved in the treatment and must give his or her consent after this information.

The basic principle for holistic healing[4] is to reverse the pathogenetic process by taking the patient into a holistic process of healing, which has been called *salutogenesis* by the great thinker Aaron Antonovsky (1923–1994)[1,2]. In the holistic clinic, this is done by giving the patient the love, support, and holding (awareness, care, respect, acceptance, and acknowledgment) that was so intensely lacking in the original traumatic events that caused the loss of inner balance, the disturbances, and the inner conflicts and was the cause of the disease for which the patient now needs healing. The trauma was caused by the repression of unbearable negative emotions and the healing must be the reverse process of the pathogenetic process according to Antonovsky. This can only happen when the patient confronts and integrates these painful emotions.

The characteristics of the state of consciousness in which patients heal (which we normally call “being in the process of healing”) have the same emotion and neural arousal as the original trauma. Because of the extreme intensity of emotions connected to certain traumas, especially from violence and sexual abuse, and especially if this happens in early childhood, it is often difficult to get these patents into the state of healing. Often lengthy therapy is needed, and patience is a must with these patients, but sometimes the therapy comes to a seemingly dead end and only more drastic and intense methods will yield the result of taking the patient into the old traumas again.

In general, what gives the holistic physician the ability to use a tool of a certain level is the complete mastery of the tools of the former steps of the staircase. Many fine books have been written on most of the techniques and level 7 and level 9 have been intensively researched, while research on the tools at level 8 has been modest. The reason for that seems obvious: both sex and violence are taboo in our culture, although they are among the best-selling commercial products (i.e., in movies and pornography). In its attempt to be clean and pure, medical science has avoided working seriously with these issues. By excluding level-8 tools and not mastering the level, the problem is that the next level becomes very difficult to handle for the therapist. Only a few contemporary therapists have used psychotropic drugs successfully, like the LSD therapy pioneer Stanislav Grof, while most often drugs have been seen as a fast route to enlightenment, the most prominent example being the drug guru Timothy Leary. More reflective people like the eminent scientist Albert Hoffmann understood perfectly well the potential of the drugs, but could not really tell how to use the drugs in therapy. The Native Americans have undoubtedly done this for years using many different drugs derived from plants and mushrooms.

The rationale for the techniques found on the therapeutic staircase (originally introduced to us by Gormsen[68]) is the most simple of all: healing happens when the present moment or now of the patient, and the old repressed and emotionally painful now, are taken together and integrated. Healing is thus the opposite of cutting your existence into parts, as you do when you repress a trauma. You heal when you, in present time, get what you could not get and needed in the past traumatic old now. So the art of holistic existential healing is really keeping the patient in the present moment, giving him or her everything needed, and at the same time taking him or her back in time into the old painful now, confronting what happened when the fundamental need was not met. If the trauma was less intense, just talking about personal history might do the job (biography work). With more intense feelings, trust and physical contact are often needed; massage is a fine example of this level 2. With more severe trauma, such as neglect in early childhood, reparenting is necessary, giving the patient the care needed, but not received, in childhood (level 3). Level 4 takes care of deeps wounds in the existence, so this is the first level of holistic existential healing. It involves a mysterious dimension of intent, and all higher levels of healing work is dependent on this. Often the trauma happened in a group setting, taking us to the logic of level 5: working with the patient in a group, recreating the sound family, healing trauma from dysfunctional families. Level 6 is rehabilitating the character and purpose of life (the soul); these deeper layers of existence are often already wounded while in the womb and without the art of deep coherence between physician and patient, allowing for an energetic imitation of the connection of the fetus and his/her mother, these wounds cannot be healed. Level 7 takes care of the body and of deep and early wounds in sexuality and gender. Level 8 integrates trauma with severe sexual and violent abuse. Direct sexual stimulation can be necessary to awaken a deeply repressed sexuality, although we strongly recommend that a patient is not stimulated into orgasm to avoid the risk of the relationship turning into a sexual relationship. Level 9 awakens the deepest layers of consciousness; the psychotropic drugs destabilize the old patterns of perception making a breakthrough possible, where the patient leaves a mental survival perspective (being in the head) to experience life fully. This project has been described as “no mind” by the Zen Buddhists. The holistic physician only uses level 10 in exceptional cases: when nothing else has worked or when time or other serious conditions do not allow for trying many different things, i.e., with terminal and suicidal patients. Direct sexual involvement with the patient will often harm the patient and cannot be recommended.

The concept of controlled violence is somewhat disturbing and it is very important that the “beating” is done only with open hand and extremely carefully, symbolically. Our team has researched the ethical problems using the holistic medical tools in general and especially the level-8, -9, and -10 techniques intensively in the last 3 years. The tool of controlled violence is highly efficient to provoke anger in patients who are so damaged by violent abuse that they are no longer able to feel and express anger. However, it is difficult to avoid strong transferences of the therapist being the violator instead of the original perpetrator from the patient’s past, making controlled violence a very dangerous tool to use for the therapist if the patient chooses to complain. Most often, the patients in need for this tool will be severely repressed and they often live in chronic fear or complete emotional numbness, socially isolated from the world. The danger of this treatment is obviously not to get them sufficiently into healing to be whole and well functioning, but sufficiently into their old material to be projecting the anger towards the therapist, and in the worst case, complain or even suing you for malpractice. Other unwanted side effects are, in rare cases, temporary psychotic episodes that are normally followed by recovery within hours; happily such episodes do not seem correlated to any negative effects of the treatment. Having legal systems in most countries that are not accustomed to the level-8, -9, and -10 techniques make the use of these tools somewhat difficult. We must recommend that you always comply rigidly with the laws of your country to avoid compromising yourself or holistic medicine in general.

## CONCLUSION

The patient will be able to heal when old painful moments, the gestalts or trauma, are taken into the present and integrated. The holistic physician has many different tools to induce this kind of healing, some of which are more dangerous and potentially traumatic than others. Using the less-powerful tool is of utmost importance to live up to Hippocrates' principle of "first do no harm". The more intense the therapeutic technique, the more emotional energy will normally be contained in the session and the higher the risk for the therapist to lose control or lose the patient to his or her own dark side, which can make the therapeutic session very traumatic and damaging to the patient.

The concept of "stepping up" in the therapy by using more and more "dramatic" methods to get access to repressed emotions and events has led to the common notion of a "therapeutic staircase" with still stronger, more efficient, and more potentially dangerous traumatic methods of therapy (see Fig. 1). This advanced expanded toolbox contains powerful tools that can be organized into a staircase of the intensity of the therapeutic experience, both according to the effect they provoke and the level of expertise they take to master.

We believe that the systematic use of the staircase will greatly improve the power and efficiency of the holistic physician and encourage governments and medical communities to work to make the whole toolbox legal for the holistic physicians of their country, as many physicians are not free to use it today, which is sad for the many chronically ill patients desperately needing more efficient holistic therapy. We hope that eventually even patients with metastatic cancer and similar diseases will benefit from the use of the advanced holistic medical toolbox.

## ACKNOWLEDGMENTS

This study was supported by grants from IMK Almene Fond. The quality of life research was originally approved by the Copenhagen Scientific Ethical Committee under number (KF)V.100.2123/91.

## BIOSKETCHES

**Søren Ventegodt, MD,** is the director of the Nordic School of Holistic Health and Quality of Life Research Center in Copenhagen, Denmark. He is also responsible for a Clinical Research Clinic for Holistic Medicine in Copenhagen and is a popular speaker throughout Scandinavia. He has published numerous scientific or popular articles and a number of books on holistic medicine, quality of life, and quality of working life. His most important scientific contributions are the comprehensive SEQOL questionnaire, the very short QOL5 questionnaire, the integrated QOL theory, the holistic process theory, the life mission theory, and the ongoing Danish Quality of Life Research Survey, 1991–94 in cooperation with the University Hospital of Copenhagen and the late professor of pediatrics, Bengt Zachau-Christiansen, MD, PhD. E-mail: ventegodt@livskvalitet.org. Website: www.livskvalitet.org

**Birgitte Clausen, RN,** leader of a secluded ward for people with dementia. Teacher at Nordic School of Holistic Medicine. Dementia coordinator and consultant in Aarhus, Denmark with more than 20 years of experience in adult psychiatry and dementia. During the last 4 years, she has worked together with Søren Ventegodt on the "QOL as medicine project" and on developing existential holistic group therapy. Through her articles and lectures, she has disseminated "The New Culture of Dementia" in both Denmark and Japan. She has participated in the production of several videos to order to inspire relatives, medical staff, and volunteers to understand people with dementia. This project has been granted financial support from the Ministry of Social Affairs in Denmark. She has conducted the first Danish investigation on the effect of sufficient daylight on the human spirit, activity level, and biological rhythm. E-mail: villahvide@mail.dk

**May Lyck Nielsen, OT,** occupational therapist from the Quality of Life Research Center in Copenhagen, Denmark. E-mail: mln@livskvalitet.org

**Joav Merrick, MD, DMSc,** is professor of child health and human development affiliated with the Center for Multidisciplinary Research in Aging, Zusman Child Development Center, Division of Pediatrics and Community Health at the Ben Gurion University, Beer-Sheva, Israel; the medical director of the Division for Mental Retardation, Ministry of Social Affairs, Jerusalem; and the founder and director of the National Institute of Child Health and Human Development. Dr. Merrick has numerous publications in the field of child health and human development, rehabilitation, intellectual disability, disability, health, welfare, abuse, advocacy, quality of life, and prevention and received the Peter Sabroe Child Award for outstanding work on behalf of Danish Children in 1985 and the International LEGO-Prize ("The Children's Nobel Prize") for an extraordinary contribution towards improvement in child welfare and well-being in 1987. E-Mail: jmerrick@internet-zahav.net. Website: www.nichd-israel.com
.
